# “HealthKick”: Formative assessment of the health environment in low-resource primary schools in the Western Cape Province of South Africa

**DOI:** 10.1186/1471-2458-12-794

**Published:** 2012-09-17

**Authors:** Anniza de Villiers, Nelia P Steyn, Catherine E Draper, Jean M Fourie, Gerhard Barkhuizen, Carl J Lombard, Lucinda Dalais, Zulfa Abrahams, Estelle V Lambert

**Affiliations:** 1 Chronic Diseases of Lifestyle Unit, Medical Research Council (MRC), Tygerberg, South Africa; 2 Centre for the Study of Social and Environmental Determinants of Nutrition, Population Health, Health Systems and Innovation, Human Sciences Research Council, Cape Town, South Africa; 3 University of Cape Town/MRC Research Unit for Exercise Science and Sports Medicine Sports Science Institute of South Africa Boundary Road Newlands, Cape Town, South Africa; 4 Western Cape Department of Education, Cape Town, South Africa; 5 Biostatistics Unit, MRC, Tygerberg, South Africa; 6 Previously from the Heart and Stroke Foundation of Southern Africa, Cape Town, South Africa

**Keywords:** Non-communicable diseases, School health environment, Nutrition, Physical activity

## Abstract

**Background:**

This study evaluated the primary school environment in terms of being conducive to good nutrition practices, sufficient physical activity and prevention of nicotine use, with the view of planning a school-based health intervention.

**Methods:**

A sample of 100 urban and rural disadvantaged schools was randomly selected from two education districts of the Western Cape Education Department, South Africa. A situation analysis, which comprised an interview with the school principal and completion of an observation schedule of the school environment, was done at all schools.

**Results:**

Schools, on average, had 560 learners and 16 educators. Principals perceived the top health priorities for learners to be an unhealthy diet (50%) and to far lesser degree, lack of physical activity (24%) and underweight (16%). They cited lack of physical activity (33%) and non-communicable diseases (NCDs; 24%) as the main health priorities for educators, while substance abuse (66%) and tobacco use (31%) were prioritised for parents. Main barriers to health promotion programmes included lack of financial resources and too little time in the time table. The most common items sold at the school tuck shops were crisps (100%), and then sweets (96%), while vendors mainly sold sweets (92%), crisps (89%), and ice lollies (38%). Very few schools (8%) had policies governing the type of food items sold at school. Twenty-six of the 100 schools that were visited had vegetable gardens. All schools reported having physical activity and physical education in their time tables, however, not all of them offered this activity outside the class room. Extramural sport offered at schools mainly included athletics, netball, and rugby, with cricket and soccer being offered less frequently.

**Conclusion:**

The formative findings of this study contribute to the knowledge of key environmental and policy determinants that may play a role in the health behaviour of learners, their parents and their educators. Evidently, these show that school environments are not always conducive to healthy lifestyles. To address the identified determinants relating to learners it is necessary to intervene on the various levels of influence, i.e. parents, educators, and the support systems for the school environment including the curriculum, food available at school, resources for physical activity as well as appropriate policies in this regard.

## Background

Chronic non-communicable diseases (NCDs) are by far the leading cause of death in the world [1]. South Africa is no exception and is considered to be in the midst of a “profound health transition” that is characterised by a quadruple burden of communicable, non-communicable, perinatal and maternal- and injury-related disorders [2]. The burden of NCDs is increasing in urban and rural settings, but most profoundly in the urban poor [2]. The prevalence and burden of type 2 diabetes is of particular concern and has been referred to as an “epidemic” faced by African countries [3].

Clearly, the health sector alone cannot bring about population-wide changes in moving toward healthier lifestyles. In this regard, the education sector has been shown to provide an effective setting for promoting healthy nutrition and physical activity behaviours in the prevention of NCDs [4]. In view of the high rates of childhood overweight and obesity worldwide, including South Africa, aiming prevention at a younger age group is essential in primary prevention [5]. Efforts to change the norms, habits, attitudes and preferences of children are still possible at a younger age, and are likely to track into adulthood [6].

Intervening in schools in disadvantaged settings in South Africa has many challenges [7]. Even after 16 years of democracy, huge disparities still exist among schools in previously disadvantaged communities compared with those in more affluent areas. Schools in disadvantaged areas usually have little access to resources; have inadequate facilities [8] and poor parent involvement [9]. Even worse, educators and learners frequently have poor motivation levels with regard to trying new interventions to influence behaviour change [10,11], and educators may feel inadequately prepared regarding curriculum changes which incorporate physical activity and nutrition [11]. There are, however, examples of school interventions which have been effective in disadvantaged settings. For example, Pathways, a randomised controlled trial to prevent obesity in American Indian school children, managed to produce significant positive changes in fat intake and food and health-related knowledge and behaviours [12].

Both the World Health Organization (WHO) [1] and the American Centre for Disease Control (CDC) [13] have provided meaningful guidelines to promote health through the school system. One of the “core elements” of the guidelines proposed by the WHO is to conduct a situational analysis prior to intervention. Such an analysis provides a baseline of factors relating to the school environment and school policy which are likely to impact on the health of the educators, learners, parents, and the surrounding community. A situation analysis helps “to better understand the needs, resources and conditions that are relevant to planning interventions” [1].

Informed by these guidelines, we undertook a situational analysis of the school health environment at 100 randomly selected primary schools in disadvantaged settings in two education districts of the Western Cape Province. One of the “major themes” of formative research is appropriateness [14] and as such one of the objectives of the situational analysis was to gain an understanding of the primary school environment of the selected schools. In this instance the health focus was on nutrition, physical activity and tobacco use. Our long-term aim was to gather information about the related environment and policies in order to develop a relevant school-based intervention programme [15]. This information was essential to construct the framework for the subsequent intervention mapping [16] used to develop the intervention. Another objective was to inform the purposive selection of 16 schools for the implementation of the HealthKick intervention. Other components included in our formative assessment were surveys of the health risk factors in educators from the 100 participating schools and another among parents of learners from those schools selected for the intervention. Only the results of the situational analysis are reported in this paper.

## Methods

### Study design

In 2007, two education districts (one rural and one urban as defined by Department of Education) in the Western Cape Province were purposively selected as the study area. Selection criteria included schools from low-income areas within each district and having at least ten Grade 4 learners per school. In each district, those schools meeting the inclusion criteria for size were stratified by the three lowest socio-economic quintiles. The quintile in which a South African school is placed depends on a score that reflects the poverty level of the community where it is located [17]. This poverty score is based on a pre-determined formula and regulates the amount of funding the school receives. This score takes into account weighted household data on income dependency ratio (or unemployment rate), and the level of education of the community (or literacy rate) as reflected in national census data [17]. Quintile one (Q1) is the poorest quintile and quintile five (Q5) the least poor. The total number of eligible schools was 243 of which 159 rural (43 Q1, 31 Q2, 85 Q3) and 84 urban (33 Q2, 51 Q3). There were only five strata, since the urban district had no schools in Q1. The sample size calculation of 100 schools for the situational analysis was based on having the precision of the 95% confidence interval for a population percentage of 50% to be 10% or less.

The number of schools selected in the five strata was proportional to the number of Grade 4 learners in the strata. Schools were randomly selected within each stratum.

### Questionnaire and observation schedule

The situational analysis comprised a structured interview with key informants (the principal or delegated person, Table 1) at each school and an observation schedule used to note specific aspects of the environment. To standardize data collection, seven fieldworkers followed a rigorous training process.

**Table 1:** General and physical environment of participating schools (n = 100 schools)

**Characteristics**	**Percentage/number (%/n)**
**Key informant**	
Principal	78
Deputy principal	11
Educators/other	9
**Average number of learners and educators**	
Learners	560
Educators	16
**Language of instruction**	
Afrikaans	73
English	6
Afrikaans and English	17
Other	4
**Home language**	
Afrikaans	89
English	3
IsiXhosa	8
**School category according to socio-economic status of surrounding communities**	
Quintile 1	22
Quintile 2	33
Quintile 3	45

The questionnaire used in the structured interview was based on relevant categories of the CDC’s School Health Index [18] and included sections on school health policy environment, physical education and other physical activity programmes, the food and nutrition environment, school health services, school psychological, and social services, health promotion for staff, family and community involvement. Development of the final questionnaire took place in close collaboration with the two senior district managers from the Department of Education.

One important aspect covered in the questionnaire included the identification of health priorities for learners, educators and parents as perceived by the principals. To engage the principals with this aspect of the questionnaire, they were shown a deck of picture cards numbered from one to eight reflecting health problems ranging from “tobacco use” to “health problems related to issues of sexuality, *e.g.* HIV and teenage pregnancies”. They were then asked to select and rank three cards representing the most pressing health problems which they thought faced learners in the school. This process was repeated for educators and parents. Various aspects about the school policy and environment relating to dietary intake and physical activity were asked, as were questions on cigarette smoking, since the prevalence of smoking among South African youth is very high [19].

Fieldworkers completed an observation schedule that was developed to align and support the interview with key respondents. This included observations of the school physical environment, i.e. safety features, facilities available, fencing, signage, hygiene, food preparation, food service, canteens or school shops, as well as physical activity and education facilities and equipment.

### Ethical approval

The study was approved by the Research Ethics Committee of the Faculty of Health Sciences, University of Cape Town (Ref no. 486/2005) and adhered to the guidelines of the Declaration of Helsinki [20] and abided by the laws of South Africa. Approval for the research was obtained from the Western Cape Education Department and school principals gave written informed consent before being interviewed.

## Results

### General physical environment of the schools

Table 1 provides an overview of the general school environment. Schools on average had 560 learners and 16 educators, with Afrikaans as the predominant spoken language. Importantly, all schools had electricity, tap water and flush toilets, although those for learners were not always ideally hygienic. Twenty-nine schools had a designated smoking room for staff. Most school buildings (89%) were in good condition, with even more enclosed by a fence and gate. Table 2 provides more detail on the physical environment of the schools. Interesting observations were that the condition of the bathrooms was frequently best in Q1 schools although their playgrounds were poorer in terms of available cemented areas, as most consisted of sand.

**Table 2:** General health environment of participating schools (n = 100 schools)

**Schools**	**Quintile 1**	**Quintile 2**	**Quintile 3**	**Total**	**CI**	*P*value*
**Condition of building**	**22**	**33**	**45**	**100**		
Neat	100	91	87	91	[87,93]	0.0102
Condition good	96	82	91	89	[85,92]	0.0460
Need a coat of paint	100	88	85	89	[84,93]	0.0240
**Condition of bathrooms**	**22**	**33**	**43**	**100**		
Soap in the bathrooms (male)	14	9	0	6	[3,10]	0.0029
Soap in the bathrooms (female)	23	9	21	8	[6,14]	0.0035
Bad smell in the bathrooms (male)	64	64	55	59	[52,66]	0.3519
Bad smell in the bathrooms (female)	23	46	31	34	[27,41]	0.0377
Towels in the bathrooms (male)	5	9	0	4	[2,8]	0.0175
Towels in the bathrooms (female)	5	9	0	4	[2,8]	0.0175
Drains blocked (male)	5	12	2	6	[4,10]	0.0329
Drains blocked (female)	5	6	4	5	[3,10]	0.8726
**Condition of playgrounds**	**22**	**33**	**45**	**100**		
Mostly grass	23	57	62	52	[45,59]	0.0001
Mostly sand with stones	77	41	27	42	[36,49]	0.0000
Cemented areas available	14	49	56	44	[38,51]	0.0000
Generally free of glass and other dangerous objects	59	85	73	74	[67,79]	0.0111
Some glass and other dangerous objects	0	12	9	8	[5,13]	0.0371
Clean of litter	0	3	2	2	[1,5]	0.4476
**Condition of sport fields**	**14**	**21**	**30**	**65**		
Grass, clean and good condition	14	15	27	20	[13,30]	0.1194
Sand/stone/muddy/litter/weeds	7	12	13	12	[7,20]	
Grass/field with posts	14	11	3	8	[4,16]	
Grass no posts	0	12	40	34	[1,12]	
Sand/gravel with posts + long grass	29	11	7	13	[7,22]	
Uneven, long grass/no posts – sand/bad condition/weeds/mole hills	29	29	40	34	[25,44]	
No space/no sports field/not suitable to use for rugby	7	6	3	5	[2,14	
Mostly grass towards ends, mostly sand with small stones	0	4	7	4	[2,11]	
**Signage**	**22**	**33**	**45**	**100**		
Sponsored by a food or beverage company	73	61	60	63	[56,70]	0.3370
**Posters**	**22**	**33**	**44**	**99**		
Physical activity	23	9	9	12	[8,18]	0.0582
Nutrition	41	36	20	30	[24,63]	0.0289

* Pearson Chi-square test adjusted for the survey design.

Over 60% of the schools displayed a signage board with the school’s name advertising a food/beverage company (Table 2), 85% (n = 54) of these were sponsored by a well-known soft drink beverage company. Principals at these schools indicated that they did not benefit financially by displaying the sponsored name boards. By comparison, posters relating to health promotion were seen at relatively few schools, and included 30 posters on healthy eating and 12 on physical activity.

When questioned about poverty, crime and violence, 84 principals indicated that they were extremely concerned about poverty and unemployment in their neighbouring communities (Table 3). Another 41% was equally concerned about crime and violence in their community. If the data are viewed in terms of the five strata (see Table 3) it is clear that principals from schools in the two urban strata were significantly more likely to view poverty and unemployment in the community as problematic. They were also more likely to view crime and violence as a problem in the school and the surrounding community. Disturbingly, more than a third of informants expressed great concern about their learners being exposed to child abuse and neglect, with significantly less in Q1 schools (14%) compared to those in Q2 and Q3 (43%, p < 0.001).

**Table 3:** Principals who expressed great concern about poverty and crime in their surrounding communities

	**Strata**							
	**1**	**2**	**3**	**4**	**5**			
**Concerns**	**Q1 Rural (n = 22)**	**Q2 Rural (n = 13)**	**Q2 Urban (n = 20)**	**Q3 Rural (n = 15)**	**Q3 Urban (n = 30)**	**Total (n = 100)**	**CI**	*P*value*
Poverty and unemployment in the community	73	62	95	87	93	84	[78,89]	0.0034
Crime and violence in school environment	0	0	20	67	33	15	[11,20]	0.0000
Crime and violence in community	9	31	70	20	60	41	[35,48]	0.0000
Child abuse/Child neglect	14	39	45	20	53	36	[30,43]	0.0002

* Pearson Chi-square test adjusted for the survey design.

### Health and health-related priorities and programmes

Table 4 shows principals’ perceptions about the health priorities for learners, educators and parents. They were asked to rank the top three health problems for each of these groups. Principals perceived an unhealthy diet (50%) and to a far lesser degree, lack of physical activity (24%) and underweight (16%) as the top three health priorities for learners. They cited lack of physical activity (33%) and NCDs (24%) most often as main health priorities for educators. Physical activity was also selected most often as the second priority (23%), while overweight (22%) ranked third. For parents, substance abuse (66%) and tobacco use (31%) were cited as the two main health priorities, respectively.

**Table 4:** Principals’ perceptions of the health priorities for learners, educators and parents

	**Top priority n = 100**			**Second priority n-100**			**Third priority n = 100**		
	**Learners % [CI]**	**Educators % [CI]**	**Parents % [CI]**	**Learners % [CI]**	**Educators % [CI]**	**Parents % [CI]**	**Learners % [CI]**	**Educators % [CI]**	**Parents % [CI]**
Tobacco use	8	9	16	14	13	**31**	10	3	10
	[5,13]	[6,14]	[11,22]	[10,20]	[9,19]	[3,4]	[6,15]	[1,6]	[7,15]
Substance abuse	15	0	**66**	9	2	19	7	4	6
	[10,21]		[59,72]	[6,14]	[1,5]	[14,25]	[4,12]	[2,8]	[3,10]
Lack of physical activity	11	**33**	3	**24**	**23**	8	15	11	10
	[7,16]	[27,40]	[1,7]	[18,31]	[17,30]	[5,13]	[10,21]	[7,16]	[6,15]
Unhealthy diet	**50**	12	7	18	19	19	6	11	17
	[43,57]	[8,18]	[4,11]	[14,25]	[15,26]	[15,26]	[4,11]	[8,17]	[12,23]
Overweight	0	12	0	1	10	1	2	**22**	4
		[8,18]		[0,4]	[7,15]	[0,4]	[1,5]	[17,28]	[2,8]
Underweight	12	0	0	2	0	0	**16**	0	1
	[8,17]			[14,25]			[11,22]		[0,4]
Chronic non-communicable diseases	0	24	3	0	18	5	7	15	14
		[19,30]	[1,7]		[13,24]	[3,9]	[4,11]	[11,21]	[10,19]
Problems related to sexual health	2	0	2	0	0	13	7	1	**25**
	[1,5]		[1,5]			[9,19]	[4,12]	[0,3]	[19,32]

Figures in bold reflect the most often selected health problems per group.

When asked about structured (formal) health promotion programmes at their schools, 52 principals indicated that these were in place. However, nearly half of them considered the national school nutrition programme (NSNP) [21] of the Department of Education (DOE) to be a structured programme. All Q1-Q3 schools participate in the NSNP, which aims to provide learners with one meal per day at school [21]. This generally comprises a starch, protein dish and vegetable/s. They mentioned that the NSNP was the most successful structured programme, since educators noticed a positive difference in learners’ behaviour as a result of receiving meals.

Once-off health promotion events were presented at most schools (89%), and largely related to foetal alcohol syndrome awareness [FasFacts programme (30%)]; HIV/AIDS (70%); and safety (77%). Nearly 85% of principals indicated that they would like to implement health promotion programmes at their school. The type of programme most frequently mentioned as being desired, related to living a healthy lifestyle within the constraints of a disadvantaged environment. Reasons for not having more structured health promotion programmes were ranked according to importance. Barriers cited most frequently were lack of financial resources and too little time in the timetable (Table 5).

**Table 5:** Main three barriers to establish health promotion programmes at schools as described by the school principals

	**1**^**st **^**Choice**		**2**^**nd **^**Choice**		**3**^**rd **^**Choice**	
	**% [CI]**		**% [CI]**		**% [CI]**	
Too little time	24	[18, 31]	5	[3, 10]	2	[0, 1]
Competing priorities	11	[7, 16]	15	[11, 21]	8	[1, 13]
Lack of human resources	12	[8, 17]	13	[8, 19]	10	[6, 15]
Lack of financial resources	21	[16, 27]	26	[20, 33]	24	[19, 31]
Inadequate facilities	14	[9, 20]	17	[13, 23]	21	[16, 28]
Lack of interest from outside bodies	3	[1, 7]	3	[1, 6]	12	[8, 18]
Lack of interest from learners	1	[0. 6]	2	[1, 5]	0	
Lack of interest from educators	0		1	[0,4]	2	[0, 5]
Lack of interest from parents	5	[2, 9]	9	[6, 14]	13	[9, 19]
Unsafe for learners to stay after school	7	[4, 11]	5	[3, 9]	2	[0, 5]

### Policy environment

Most of the schools (89%) that were surveyed had a health and safety committee, which mainly dealt with safety issues, while 14% also dealt with nutrition-related issues and 1% with physical activity. Only 33% of schools indicated that there was substantive parental involvement in the health and safety committee. Furthermore, nearly half of the principals indicated that the school governing body (SGB; parents and other stakeholders) played a slight or no role in the management of the school. Most principals (76%) indicated that their schools were accessible to the community after school hours, mainly for religious activities (62%). A very small number indicated that their facilities were used for sport and sport-related activities (13%).

Only eight percent of the schools with tuck shops (a school shop mostly selling snack items) had a school governing policy for operating purposes, while 37% of schools with mobile vendors providing food and snack items to the learners had a policy guiding these activities (Table 6). Overall, less than 12% of the schools had policies relating to food that was allowed at functions and outings.

**Table 6:** Type of policies found at schools and compliance with these

**Policies**	**Schools (%)**	**Confidence Interval**	**Compliance (%)**
Tuck shop	8	[5, 13]	28
Vendors	37	[31, 44]	32
Lunchbox	8	[5, 13]	7
Feeding scheme	51	[44, 58]	51
Food as reward	4	[2, 8]	11
Food at outings	9	[6, 14]	9
Food for fund-raising	11	[7, 17]	11
Food at events	12	[8, 18]	10
Smoking-learners	95	[91, 97]	-
Smoking-staff	86	[80, 90]	89
Smoking-visitors	82	[76, 87]	89

With regard to tobacco use, most schools had a policy for learners, their staff and for visitors. Concerning adherence to these policies, 89% of principals indicated that they complied with the policy inside school buildings and vehicles, 76% complied outside school buildings and 72% at school events away from the school.

### Nutrition environment at the schools

Table 7 indicates that learners were able to purchase food from a tuck shop at 64% of schools, while those at 33% of schools purchased food items from vendors outside the school grounds and 6% at a shop close to the school. In South Africa, mobile school vendors (usually women) frequently sell low-cost food items through the school fences, since they are usually not allowed on the school premises. Tuck shops were mostly managed by educators (58%) though some parents and the SGB (25%) also undertook this role. Principals in general thought that the tuck shops were reasonably well supported and most schools benefitted from the profit made on sales. Decisions about what items to sell at the tuck shop were often done by educators (67%) and seldom by learners (8%).

**Table 7:** Reported and observed data regarding food provided/sold at the 100 schools

**Variables**	**Q1 % [CI]**	**Q2 % [CI]**	**Q3 % [CI]**	**Total % [CI]**	*P*value*
Schools with tuck shops	59 [44,73]	51 [40, 63]	76 [65, 84]	64 [57, 70]	0.0078
Vendors on property	0	62 [3, 14]	9 [5, 16]	6 [4, 10]	0.596
Vendors outside school fence	18 [9, 33]	31 [22, 41]	42 [32, 53]	33 [27, 40]	0.0146
Shop near school	5 [1, 17]	3 [0, 10]	8 [4, 18]	6 [3, 11]	0.2913
National School Nutrition Programme	100	97 [90, 99]	98 [92, 99]	98 [95, 99]	0.4476

* Pearson Chi-square test adjusted for the survey design.

The most common items sold at the tuck shops were crisps (100%), sweets (96%), cold drinks (41%), ice lollies (41%), and chocolates (28%). The most common items sold by vendors were sweets (92%), crisps (89%), ice lollies (38%), fruit (25%), doughnuts (21%), hot dogs/burgers (13%), and fat cakes (fried dough balls) (11%).

Twenty-six of the 100 schools that were visited had vegetable gardens. The most commonly mentioned purpose was to supplement the NSNP meals. The main reasons provided for schools not having a vegetable garden centred on lack of space, the grounds being unsuitable, and a lack of time.

### Physical activity environment

Principals from all schools indicated that structured physical activity lessons or the physical development and movement (PDM) outcome in the Life Orientation curriculum were scheduled in the weekly timetable. Schools had mostly one or two sessions scheduled, as shown in Table 8. In the Foundation Phase, less than 60% and in the Senior Phase less than 50% of principals indicated that this translated to learners actually participating in physical activity outside the classroom.

**Table 8:** Physical activity (PA) in the time table and actual PA sessions outside the classroom

	**No sessions (%)**	**One session (%)**	**Two sessions (%)**	**Other (%)**
PA in the time table				
Foundation Phase	0	59	40	1
Intermediate Phase	0	55.4	42.6	1
Senior Phase	0	47.5	48.5	
PA sessions outside the classroom				
Foundation Phase	41	22	22	5
Intermediate Phase	43.6	27.7	19.8	9
Senior Phase	51.5	24.8	14.9	8

All schools offered extramural sport. Most of the schools participated in athletics (n = 94), netball (n = 93), and rugby (n = 81), while 76 offered cricket and only 59 soccer. Only a few of these schools seemed to have had adequate equipment for these sports with only a few balls observed at most of the schools that offered netball and rugby (n = 86 and n = 76 respectively). The higher quintile schools were more likely to offer netball, cricket and soccer than the lower quintile schools, although the frequency of athletics and rugby offered did not differ across quintiles. Sports, other than those mentioned above, were seldom played because of lack of equipment or lack of facilities.

Only 19% of the principals indicated that their schools’ sport facilities were adequate, while the remainder indicated that their facilities needed upgrading. This perception was confirmed by the observational data showing an average of 1 netball court and 1 grass field per school. Only nine netball courts had field markings and posts and only 13 grass fields were in a good condition. The equipment available at schools was mainly provided by parents or the community and to a lesser degree by the DOE, while educators were the main coaches. Recreational playing during break time involved chasing each other, playing ball or skipping, however, very few schools made equipment such as balls or skipping ropes available to learners. In 94% of the schools, educators were allocated to supervising break times and their presence was confirmed by the observational data.

### Tobacco use

Although 86% of principals reported that none or very few of their learners smoked it must be noted that they rated smoking as the second most important health priority among parents. Thirteen percent of schools indicated that many learners were seen smoking on the school grounds, while only 1% of educators was reportedly seen smoking on school grounds.

## Discussion

The aim of the situational analysis was to provide a baseline assessment of the environment in low-income primary schools relating to nutrition, physical activity and smoking behaviour. This would assist in designing an effective school-based intervention programme to prevent NCDs.

From the observational data, the general physical environment of the schools appeared to be in good condition, although concerns existed around the hygiene of toilet and bathroom facilities. This could have implications for hand hygiene, since the three primary measures associated with reduced incidence of infections include availability of a clean water supply, adequate disposal of waste (particularly for faeces), and hand hygiene [22].

Another aspect of the general environment which needs addressing is the large number of signage boards on school premises sponsored by a soft-drink company. Displaying these boards would imply that the school supports and promotes the use of these particular soft drinks. Clearly this is not a health promotion message that should be encouraged, a view which is supported by findings from a qualitative study conducted in Australia with primary school learners [23]. This study found that children appeared to believe that school, and anything permitted at school, is inherently healthy. Further support came from parents, who postulated that the inconsistent messages about unhealthy energy-dense foods, including attractive marketing and advertising strategies, confused children [23].

The data gathered from a large percentage of the principals provided insight into the surrounding communities. Principals expressed their concerns regarding the existence of poverty, unemployment, crime, violence and especially child abuse in these communities. Principals from the urban district identified their learners were facing these problems to a greater extent than the principals from rural schools. Clear differences were observed in the perceptions of urban and rural district principals that were not linked to the poverty grade (quintile). These findings are interesting because poverty is, as elsewhere in South Africa, a rural phenomenon, with the rural poverty rate in the Western Cape estimated at 26.1% compared to 20.1% in urban areas [24]. The reasons for this difference in perceptions about poverty and employment could possibly be explained by the fact that poverty and unemployment were referred to in the same question, and schools in the urban district are mostly located in the Cape Town municipality, which has the fourth highest unemployment rate in the Western Cape, despite having the lowest poverty rate [24]. The learners in these schools were, therefore, not only from communities facing high unemployment, but also from the poorest households in the urban district. On the other hand, learners in the rural district were often from agricultural households that face lower unemployment levels, although they have a higher poverty rate than their non-agricultural counterparts. The reason for the different perceptions about child abuse and neglect is less clear as it appears that living in deep poverty increases the vulnerability of children for abuse and neglect [25].

Perceptions about higher crime and violence in the urban district are supported by findings that the homicide victimisation rates for men aged 15–29 years in the Cape Town townships, where many urban schools are located, are more than twice the average for the country [26]. Furthermore, crime and violence are closely related to alcohol and substance abuse. A review of studies on substance abuse trends found that the Western Cape had the second highest (7.1%) 12-month prevalence of substance use disorders and the highest (18.5%) lifetime prevalence of substance use disorders compared to other provinces [27,28]. In South Africa, the Western Cape had the highest alcohol consumption among males and females. Therefore, it is not surprising that principals reported substance abuse to be one of the top three health priorities for parents.

Regarding health and health-related priorities and programmes, it is clear from the results that school principals considered lifestyle-related health issues to be priorities for learners, educators and parents. They also indicated a need for programmes to address these health priorities. However, various barriers to implement these were identified, with lack of time and financial resources being the most important ones. Lack of time has been raised elsewhere as being a major barrier to health promotion programmes [29,30]. A study of Intermediate Phase (grades 4–6) educators also raised time limitation as a major problem for the implementation and presentation of Life Orientation, a compulsory learning area for primary school children. This learning area was introduced as part of the outcomes-based education system implemented in 1997 to eradicate the inequalities of the apartheid education system [31]. Life Orientation comprised four learning outcomes for the Intermediate Phase, i.e. health promotion, social development, personal development, and physical development and movement [32], which made this the ideal vehicle for health promotion programmes. Changes have been made to the National Curriculum since the implementation of the HealthKick intervention. Life Orientation is now called Life Skills and is divided into three study areas: Personal and Social Well-being, Physical Education and Creative Arts [33]. At this stage, there is no clarity how external health promotion programmes, such as HealthKick, may be integrated into this subject. The WHO Health Promoting Schools (HPS) initiative however provides an existing framework for school health programmes [34]. Strengthening this initiative is furthermore listed as a strategy to address inequity and social determinants of health by the South African National Department of Health [35].

Overall, parental and community involvement appeared to be poor. Only a third of the schools had parents who were involved in the school health and safety committee. This was also reflected by a lack of involvement of the parents in the SGB and the small number of schools where parent or SGB involvement in the school tuck shop was reported. A study conducted in 2004 on parental participation in SGBs, showed that lack of participation could be attributed to the low education level of parents in disadvantaged communities, language barriers and difficulty in attending meetings [9].

The school food environment has been identified as one of the most important components in effective school-based interventions to promote healthy eating [1,36]. Apart from the finding that most of the schools participated in the NSNP, a large number had tuck shops and vendors selling food items. The findings clearly point to a need for intervention, since sweets (candy), chocolates, cold drinks and crisps were the main items sold by these providers.

Observations of learner spending during the survey showed that they spent little money at the tuck shop or food vendors. The small profit made is either used by the school to provide extra services to learners, or for vendors to make a living. This finding is supported by an evaluation report of the NSNP, which found that learners brought less than $0.3 to school to spend on food of “very poor nutritional value” [37]. The fear of losing the income (although small) generated by the sale of these items may however provide a barrier to the willingness of the school and the food vendors to sell healthier food items. An aspect that warrants further investigation, specifically in context of a developing country, has been recommended by Von Holy and Makhoane [38]. They suggest that baseline research is required to determine the safety and socio-economic importance of foods sold by vendors.

The feasibility of intervening in the nutrition-related environment of schools in disadvantaged settings where children’s buying power is limited, has received very little attention in the literature. A review on the impact of improving nutrition standards on school revenue concluded that in the North American situation very little evidence exists that revenues drop when healthier policies are adopted; however, this may not be the case in disadvantaged African settings [39].

Very few schools in this study had clear policies guiding the food, nutrition and physical activity environment. This could possibly be ascribed to such policies not being required by the DOE. The WHO [1], in their global strategy on the prevention of NCDs, urges government to draft policies that stimulate schools to promote healthy eating and encourage physical activity. Intervening in schools at the policy level has been successful in many studies [40]. In this regard South African schools are slow to follow.

Although principals from all the schools indicated that structured physical activity lessons were scheduled in the weekly timetable, this did not always translate to learners actually participating in physical activity outside the classroom. Possible explanations for this finding comes from a study in a similar sample by Van Deventer [11], where educators indicated that the reasons for not presenting the Physical Development and Movement outcome had to do with a lack of time in the curriculum, educators who were not qualified to teach physical education and a lack of facilities and equipment. The latter is also relevant for extramural sport. Generally, the results showed that extramural sport was being offered at schools but that facilities, although available, were often not sufficient and/or in good condition. These findings reflect the legacy of apartheid when sport development and participation was a privilege set aside for only a small segment of the population [9]. Inadequate facilities were found to be the most important factor for non-participation in sport by black secondary school learners [8].

Although children seem to be active and educators allocated for supervision during break times, no organised activities were observed. Leviton [41] referred to studies which showed that learners were most active when equipment and facilities, such as basketball hoops and better playgrounds, were available along with organised active games under supervision.

Lastly, tobacco use is an important preventable factor in the war against NCDs [42]. The finding that 13% of school principals reported their learners were frequently smoking on school grounds is of great concern considering their age. Findings from the 2008 South African Youth Risk behaviour survey support our results [19]. In this survey, 21% of the youth aged 13 to 18 years indicated smoking daily. Clearly schools and parents need to take additional means of preventing such behaviour. However, schools also need to address the smoking practices of educators as a substantial number of schools (n = 29) reported having smoking rooms for their staff. Although the tobacco control act [43] allowed this at the time, the negative modelling effect [44] this practice could have on learner behaviour should be considered.

## Conclusions

The formative findings provide important information about the context in which primary school learners’ health behaviour takes place in disadvantaged schools in the Western Cape. Evidently, these show that school environments are not always conducive to healthy eating, sufficient physical activity or the prevention of tobacco use. A limitation of the study is that some of the findings, such as those on health priorities within the school community, rely only on the perspective of the school principals and the views of learners, parents and educators were not obtained. The findings do however contribute to the knowledge of key environmental and policy determinants that play a role in the health behaviour of learners, their parents and educators. This knowledge is essential to create an intervention programme in the school setting when trying to optimise the physical environment and develop relevant school policies.

## Competing interests

The authors declare that they have no competing interests.

## Authors’ contributions

ADV: All aspects of the study: planning, implementation, writing up. NPS: All aspects of the study: planning, implementation, writing up. JMF: Questionnaire design and development; writing and editing final draft. CED: Questionnaire and observational schedule design and development, data collection, editing final draft. GB: Consultant: Western Cape Education Department; planning and implementation. CJL: Data analyses and interpretation. LD: Co-ordinator. ZA: Questionnaire design and development; writing up. EVL: All aspects of the study: planning, implementation, writing up.

## Pre-publication history

The pre-publication history for this paper can be accessed here:

http://www.biomedcentral.com/1471-2458/12/794/prepub
